# Inhibitory effect of coumarin and its analogs on insulin fibrillation /cytotoxicity is depend on oligomerization states of the protein†

**DOI:** 10.1039/d0ra07710k

**Published:** 2020-10-16

**Authors:** Mohsen Akbarian, Ehsan Rezaie, Fatemeh Farjadian, Zahra Bazyar, Mona Hosseini-Sarvari, Ehsan Malek Ara, Seyed Ali Mirhosseini, Jafar Amani

**Affiliations:** Molecular Biology Research Center, Systems Biology and Poisonings Institute, Baqiyatallah University of Medical Sciences P.O. Box 19395-5487 Tehran Iran rezaie.ehs@gmail.com +98 21 83062555 +98 21 82454555; Pharmaceutical Sciences Research Center, Shiraz University of Medical Sciences Shiraz Iran; Department of Chemistry, Shiraz University Shiraz Iran; Applied Microbiology Research Center, Baqiyatallah University of Medical Sciences Tehran Iran

## Abstract

Looking through a historical lens, attention to the stabilization of pharmaceutical proteins/peptides has been dramatically increased. Human insulin is the most challenging and the most widely used pharmaceutical protein in the world. In this study, the protein and coumarin as a plant-derived phenolic compound and two coumarin analogs with different moieties were investigated to evaluate the protein fibrillation and cytotoxicity. The obtained data showed that with a change in environmental pH, the behavior of the compounds on the process of insulin fibrillation will be changed completely. Coumarin (C1) and its hydrophobic analog, 7-methyl coumarin (C2), in an acidic environment, inhibit insulin fibrillation, change the oligomerization state of insulin and produce fibrils with notable lateral interactions with low cytotoxicity. However, negatively-charged 3-trifluoromethyl coumarin (C3) without significant changes in insulin structure and by altering the oligomerization state of the protein, slightly accelerates hormone fibrillation. Also, the compounds showed a disulfide protecting role during protein aggregation. Regarding the toxicity of the fibrils, it was observed that in addition to the secondary structures of proteinous fibrils, the ability to destroy the cell membrane is also related to the length of the fibrils and their degree of lateral interactions. By and large, this work can be useful in finding a better formulation for bio-pharmaceutical macro-molecules.

## Abstract 2

The effect of the applied compounds on insulin fibrillation at two pHs. By and large, the compounds through changing the oligomerization states and altering structure integrity of insulin can govern the fibrillation process.

## Graphical abstract

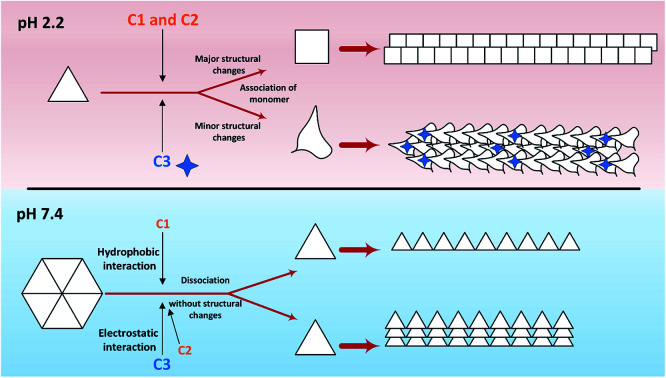

## 1. Introduction

Some proteins/peptides easily aggregate/fibrillate in the presence of environmental stresses.^1^ There are several proteinopathies such as Parkinson's and Alzheimer's diseases that have been known to have a close relationship with the aggregation of functional or structural proteins.^2,3^ Also, protein misfolding, amorphous and morphous aggregations of these molecules are known as a big challenge in the industrial production of therapeutic proteins and peptides.^1,4^ Therefore, the stabilization of these biopharmaceutical products in different ways is an important field in the world of biotechnology. Several studies have been carried out to increase the stability of therapeutic proteins. Some of the researchers used nano-mediated systems,^5–7^ others tried to use proteinous chaperons to inhibit the aggregation/fibrillation of low stable proteins.^8,9^ For reviewing all investigated foreign molecules such as nanoparticles, synthetic and plant extracted compounds and different proteins and peptides on insulin fibrillation, ref. 10 would be helpful. Among all these routes, based on centuries of coexistence between humans and plants, the use of phytochemicals for this purpose has received more attention. Along this line of strategy, the use of phenolic compounds such as curcumin,^11–13^ gallic acid,^14^ ferulic acid,^15^ quercetin,^16^*etc.* have attracted a lot of attention.

Among all studied phenolic compounds, coumarin has many beneficial properties. It is known as a backbone for a varied class of synthetic and natural compounds^17^ that exhibited multipurpose pharmacological activities containing antioxidant, anti-inflammatory,^18^ antithrombotic,^19^ antinociceptive,^20^ antiviral,^21^ hepatoprotective,^22^ antidepressant,^23^ antimicrobial,^24^ anti-carcinogenic,^25^ antituberculosis,^26^ anticholinesterase,^27^ antihyperlipidemic,^28^ and antidepressant^29^ activities. Recently, Ulicna *et al.* showed the inhibitory effects of some tacrine–coumarin heterodimers on hen egg-white lysozyme fibrillation.^30^

**Scheme 1:**
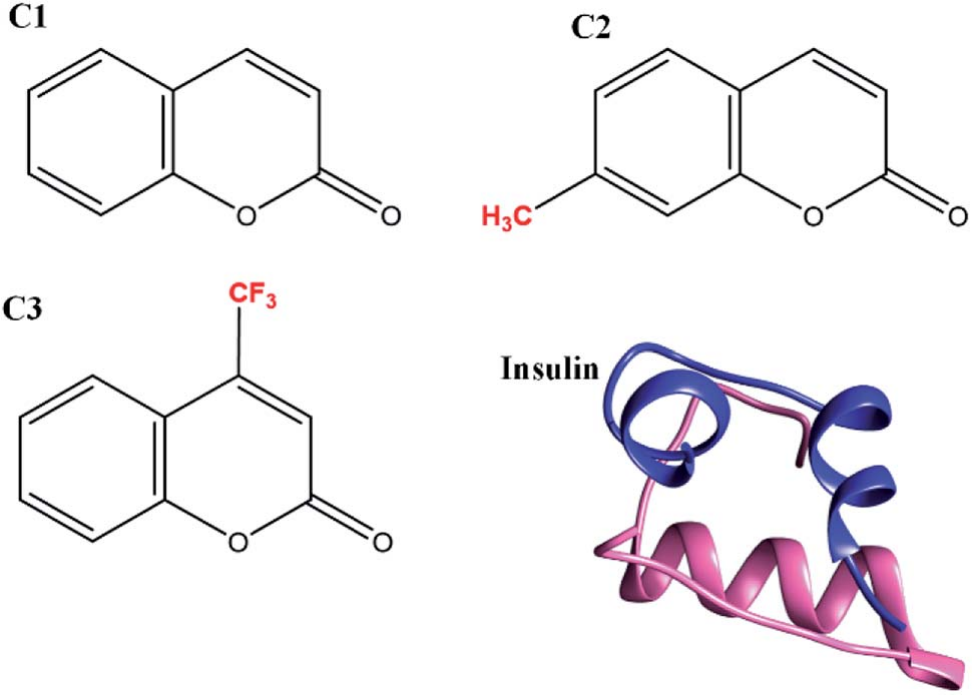
Schematic structure of studied compounds in this research. C1 stands for coumarin, C2 and C3 show 7-methyl coumarin and 3-trifluoromethyl coumarin, respectively.

To achieve the correct and ideal mechanism for protein stabilization, a closer look should be given to the types of forces and parameters involved in the stability and instability of therapeutic proteins. For effective inhibition of amyloidogenic proteins, several thoughts showed us hydrophobic or electrostatic groups are unavoidably important during the process.^5,31^ However, no general agreement indicated which one is more important than another. Herein, along with coumarin, we synthesized two analogs of the compound containing negative charges (–CF_3_) and hydrophobic (–CH_3_) groups to detect the importance of these two moieties on the fibrillation of human insulin as a well-known amyloidogenic protein model (Scheme 1).

In this study, through different physicochemical methods as well as *in vitro* cell toxicity, using coumarin and two types of its analogs that have different hydrophobic and electrostatic properties, fibril formation and toxicity of human insulin were studied.

## 2. Materials and methods

### 2.1. Materials

Bis-1-anilino-8-naphthalene sulfonate (bis-ANS), Thioflavin T (ThT), recombinant human insulin (UniProt ID P01308), 3-(4,5-dimethylthiazol-2-yl)-2,5-diphenyl tetrazolium bromide (MTT), 1,4-dithiothreitol (DTT) and other chemicals were purchased from Sigma (Aldrich, USA). Solvents used for reverse-phase high-performance liquid chromatography (RP-HPLC) were purchased from Caledon Company. Ethanol, acetone and distilled water that were used for washing were acquired from Merck Darmstadt (W. Germany). Media and solutions (Dulbecco's Modified Eagle Medium (DMEM), phosphate buffer saline (PBS), fetal bovine serum) for culturing N2A cells were obtained from Thermo Fisher Scientific (USA). Penicillin–streptomycin solution was bought from Sigma (Aldrich, USA).

### 2.2. Methods

#### 2.2.1. Spectroscopic analyses of coumarin analogs

Coumarin, 7-methyl coumarin and 3-trifluoromethyl coumarin (C1, C2 and C3, respectively) were dissolved (100 mM) in dimethylsulfoxide (DMSO), then diluted (10 μM) in 20% acetic acid (pH 2.2) containing 100 mM NaCl to mimic the fibril formation media.^31^ The stabilities of the compounds were studied by recording obtained maximum emission wavelength (*λ*_max_ emission) during 20 h intervals by a Cary Eclipse fluorescence spectrophotometer (Varian Cary Eclipse, USA) which armed with a Peltier unit to control the temperature.

#### 2.2.2. Kinetics of monomeric and hexameric states of insulin fibrillation

To induce monomeric-fibrillogenic condition for insulin (351 μM), 20% acetic acid (pH 2.2) containing 100 mM NaCl at 60 °C was considered.^31^ In the presented situation, insulin molecules transformed into monomeric units, and high temperature changes the native structure of insulin to partially unfolded states, a standard condition to study insulin fibrillation.^31^ For insulin fibrillation in neutral conditions, insulin (351 μM) was dissolved in tris buffer (25 mM, pH 7.4) containing 100 mM NaCl and 3.0 : 1.0 ratio of insulin monomer to ZnCl_2_ (Zn ion). This ratio is suitable for making insulin hexamer.^31^ The process was conducted at 37 °C with continuous shaking (760 rpm). The intensity of fluorophore ThT at 484 nm was monitored for tracking the rate of fibrillation. It has been well known that due to the binding of ThT molecule to the proteinous fibrils, the positions of excitation and emission maxima changed from 350 and 450 to 450 and 484 nm, respectively.^32^ The molar extinction coefficient of 24 420 M^−1^ cm^−1^ at 420 nm was used for the preparation of 1.0 mM ThT in double-distilled water.^5^ 10 μM ThT was set as the final concentration of the fluorophore in each insulin solution. Kinetics of the fibrillation were studied in a dark 96-microwell plate. For each well, a total solution of 150 μL was considered as the final volume. For removing the buffer contribution, in parallel for all experiments, the same solutions without insulin were prepared and their corresponded spectra were subtracted from the protein spectra. To reduce well to well variations, all samples were prepared at least triplicate and the related data were presented as their average. Curve fitting and calculating kinetic parameters were calculated by GraphPad Prism V7.0 (GraphPad Software, Inc La Jolla, California, USA). For all experiments, insulin solution at the final concentration of 2.0 mg mL^−1^, 351 μM, was prepared freshly using molar extinction coefficient of 1.08, at 276 nm, for 1.0 mg mL^−1^.

#### 2.2.3. Structural characterizations of insulin

##### 2.2.3.1. Tyr fluorescence assessment

The intrinsic fluorescence of human insulin was scanned using exciting Tyr residues at 276 nm and collecting the emitted lights at a range of 280–400 nm. Any alteration in the tertiary structure of the protein can affect the intensity of the emission light.^4,33^ To doing so, insulin (2 mg mL^−1^) was dissolved in the defined acid condition containing the indicated different concentrations of the analogs and after the considered incubation time, the sample was diluted to 1.0 mg mL^−1^ of the protein.

##### 2.2.3.2. Spectroscopic assessment of the exposed hydrophobic surfaces of insulin by bis-ANS dye

Under the acidic-fibrillogenic condition, the binding of bis-ANS molecules (10 μM, excitation and emission: 350 nm and 400–600 nm, respectively) to insulin (2 mg mL^−1^) was studied in the presence of different analog concentrations (10, 20 and 30 μM).

##### 2.2.3.3. Monitoring secondary structure of insulin using circular dichroism spectroscopy

Secondary structure contents of the protein in the different conditions (both defined acidic and neutral conditions) were evaluated by recording ellipticities (Jasco J-810 circular dichroism (CD) spectropolarimeter) in the far-UV region (260 to 190 nm). Insulin concentration was prepared at 2.0 mg mL^−1^. Like previous experiments, for eliminating the contribution of the buffers, the same solutions without the protein were prepared and the obtained blank spectra were subtracted from the test spectra. All the experiments were conducted three times and the presented data were reported as the averages of the replicates.

#### 2.2.4. Attenuated total reflectance Fourier transform infrared spectroscopy

During this study, structural polymorphisms of the preformed fibrils were investigated by attenuated total reflectance Fourier transform infrared (ATR-FTIR). Under different analog concentrations, insulin hormone (2.0 mg mL^−1^) was incubated for 10 h at the acidic-fibrillogenic condition. The produced fibrils were washed (20 min, 13 000*g* at ambient temperature) three times with D_2_O to replace any hydrogen-contain molecules and unbounded residual buffers by deuterons. Finally, the fibrils spread on the attenuated total reflectance (ATR) diamond cell (Tensor II instrument, Bruker, Germany). The board range region (4000 to 400 cm^−1^) by 2 cm^−1^ resolution and an accumulation of 256 scans were recorded for calculating the secondary structure of the preformed fibrils.

#### 2.2.5. Confocal Raman spectroscopy

Similar to what was done to prepare the fibril samples for the FTIR study, the fibers were also prepared for Raman spectroscopy. In brief, insulin (2.0 mg mL^−1^) was incubated with 30 μM of the compounds at the acidic condition for 10 h. By completing the fibril formation, the aggregated proteins were collected by centrifugation (20 min, 13 000*g* at ambient temperature). The precipitates were washed three times with D_2_O for eliminating any residual of the salt (NaCl), water and acetic acid. After lyophilization of the precipitates, a 15 mg of powder fibrils was spread uniformly on the cell surface of the device (Lab Ram HR, Horiba, Japan). A 785 nm diode laser was focused on the fibril samples with a 100× objective (0.5 NA-Olympus).

#### 2.2.6. Transmission electron microscopy

Overall polymorphisms of the generated fibrils were studied by transmission electron microscopic (TEM) imaging. For generation of the fibrils, insulin (2.0 mg mL^−1^) was dissolved in 20% acetic acid (pH 2.2) containing 100 mM NaCl. Also, insulin fibrillation at neutral condition (insulin (2.0 mg mL^−1^), tris buffer (25 mM, pH 7.4 containing 100 mM NaCl and 3.0 : 1.0 molar ration of insulin to ZnCl_2_) with continuous shaking (720 rpm)) was done for evaluating the generated fibrils in this condition. For TEM imaging, between three indicated concentrations of the compounds, the maximum amount (30 μM) was considered for fibril formation. After 10 and 30 h incubations at 60 °C and 37 °C in acidic and neutral conditions, respectively, the aggregated solutions were diluted 5 times by double distilled water and loaded on the formvar carbon-coated copper grids. After negatively staining with 1% aqueous uranyl acetate, the fibrils were observed at 100 kV excitation voltages with a Philips CM10 transmission electron microscope (Philips, The Netherlands).

#### 2.2.7. Fluorescence microscopy

To detect the obtained fibrils in both acidic and neutral solutions by fluorescence microscopy, the specimens were mixed with fluorophore ThT dye (to a final concentration of 50 μM) and spread uniformly on a microscopic slide and then incubated in a dark place for 30 min. After staining the fibrils by the dye, the instrument (Lionheart FX, BioTek, USA) was set at 469/525 nm filters (excitation/emission, respectively) and the fluorescence images were taken.

#### 2.2.8. Dynamic light scattering study

The dynamic light scattering (DLS) measurements were performed at both defined acidic and neutral conditions using a Horiba instrument (SZ-100, Kyoto, Japan). For acidic condition, the samples (2.0 mg mL^−1^ insulin in different concentrations of the compounds) were incubated at 60 °C. Freshly prepared insulin is also studied at pH 7.4 in tris buffer (25 mM containing 100 mM NaCl and 3.0 : 1.0 molar ration of insulin to ZnCl_2_) at 37 °C.^31^ Also, for zeta potential assessment of the applied compounds, 100 μM of the chemical candidates were dissolved separately in the previously defined acidic and neutral solutions and the zeta potential was measured.

#### 2.2.9. Size exclusion chromatography

Oligomerization of insulin molecule was evaluated by high-performance liquid chromatography system equipped with 300 × 8 mm PSS SUPERMA column (KNAUER, Germany). The protein (2.0 mg mL^−1^) like previous experiments was incubated in the presence of different concentrations of the compounds and then a 20 μL (40 μg insulin) of each sample was analyzed by the system using 1.0 mL min^−1^ flow rate over 10 and/or 15 min at a constant temperature. To detect the protein, absorbance at 214 nm (peptide bonds absorption) was recorded (DAD2 KNAUER UV detector). Chromatograms were obtained using the solutions, isocratically. For the acidic and neutral conditions, in the previously defined solution and buffer, temperatures of the size exclusion column (using AZURA CT 2.1 column thermostat) were set at 60 °C and 37 °C, respectively.

#### 2.2.10. Reverse phase-high performance liquid chromatography

The hydrophobicity of the compounds was assessed by the analytical KNAUER system armed with a C18 column (ProntoSIL 200-5-C18, 250 × 4.6 mm; Apex Scientific). The chromatogram was obtained by acetonitrile/water (ACN/water) 40/60 ratio isocratically over 10 min run time with 1 mL min^−1^ flow rate at ambient temperature (AZURA CT 2.1 column thermostat). The compounds (100 μg mL^−1^) were dissolved in the running solution and 20 μL of each mixture was took and loaded onto the column. The chromatograms were obtained using the *λ*_max_ absorbance of the compounds. RP-HPLC experiment was also performed to judge the hydrophobic levels of insulin (351 μM) during treatment with the synthetic compounds (30 μM). These three samples with control specimen were separately subjected to the RP-HPLC column at 25 °C. To avoid overlapping the compounds and insulin peaks, chromatograms were obtained with a flow rate of 1 mL min^−1^ and a linear gradient of acetonitrile (24–60%) for 30 min. For detecting the elution time, the UV detector was set at 214 nm (peptide bonds absorption wavelength).

#### 2.2.11. DTT induced insulin aggregation

Because of the instability of DTT in acidic condition, neutral pH (pH 7.4) was selected to conduct DTT-induced insulin aggregation. To study insulin stability against the reduction stress, 0.5 mg mL^−1^ insulin was prepared using tris buffer (25 mM) at pH 7.4, containing 100 mM NaCl and ZnCl_2_ (3.0 : 1.0 ratio of insulin monomer to ZnCl_2_). C1, C2 and C3 compounds were added into the protein solution in different indicated concentrations. After adding DTT (20 mM) into the solution, the experiment was done at 43 °C for 20 min. The kinetic of aggregation was assessed by recording the absorbance at 360 nm using T90+ UV-Vis spectrophotometer (PG Instruments, UK) equipped with a Peltier temperature controller.

#### 2.2.12. MTT reduction assay

N2A cell (a fast-growing mouse neuroblastoma cell line) were cultured in DMEM with stable glutamine in 5% CO_2_ at 37 °C. The medium contains 10% FBS and 1% penicillin–streptomycin antibiotics. To study the cell toxicity of the compounds and insulin fibrils, PBS-washed generated insulin fibrils (three times) were centrifuged (20 000*g* for 20 min) and the pellet was resuspended in 200 μL the sterile medium with different analogs concentrations. To seed the cells, 100 000 cells were loaded in each well of 96-well flat-bottom microplate and incubated for 24 h. After that, the old medium was replaced by 100 μL of the new fibril/compounds-contained medium (0.2 μg μL^−1^ of the fibrils and different concentrations of the compounds) and incubated for 24 h at the culturing conditions. Then, a 10 μL of MTT solution (5.0 mg mL^−1^ prepared in phosphate buffer saline) was added to each well and incubated for a further 4 h. Subsequently, SDS-DMF solution (100 μL in each well, 50% DMF and 20% SDS, pH 4.75) was added and kept 18 h in the defined humidified CO_2_ conditions. Using a BioTek (USA) microplate reader the absorption values were recorded at 560 nm. Suitable blank controls were considered for all test samples. By calculating 100% viability for the negative control, the result was plotted as percentage cell viability.^34–36^ All the experiments were repeated five times.

#### 2.2.13. Statistical analyses

Using GraphPad Prism software (version 7.0) and by one-way ANOVA method, the significance was statistically analyzed. All the presented data were shown as mean ± SEM and using analysis of variance the statistical significance between the groups was determined. *P* < 0.05 was considered to be statistically significant.

## 3. Results

### 3.1. Structural changes of insulin in the presence of coumarin and its analogs

**Fig. 1:**
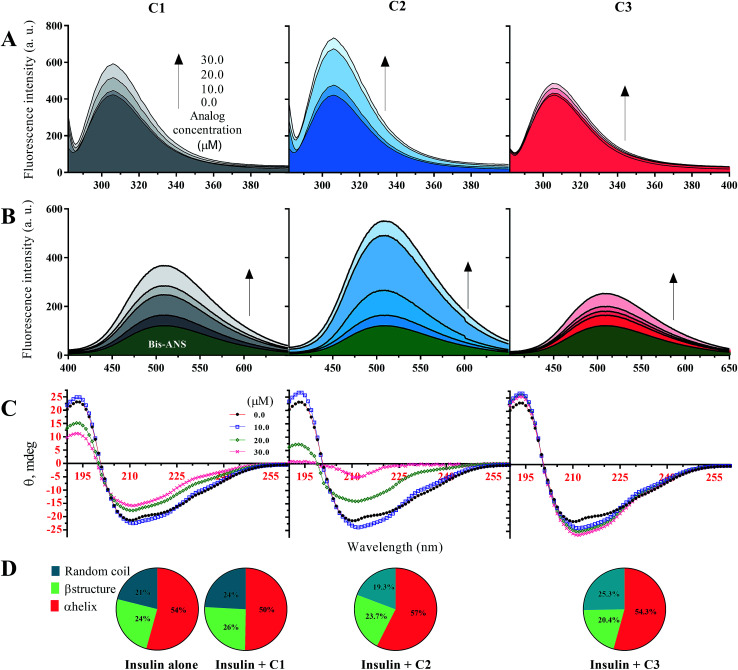
Structural changes of insulin in the presence of C1, C2 and C3 in acidic (pH 2.2) and neutral (pH 7.4) conditions. (A) indicates intrinsic fluorescence changes of insulin in the presence of the compounds at different concentrations. Using bis-ANS fluorophore dye for monitoring hydrophobic surface, surface hydrophobicity of the protein in the presence of the compounds was examined (B). The effects of different concentrations of the considered compounds on secondary structure contents of insulin were studied (C). Pie charts (D) indicate deconvoluted (estimated using CDNN software)^31^ CD spectra of insulin in the presence of the compounds (30 μM). The (A–C) were done in an acidic environment (pH 2.2) while (D) was done at neutral pH (pH 7.4). All the spectra were recorded three times and indicated as average.

In this study, before examining the fibrillation of insulin in the presence of the studied compounds, secondary and overall structural changes of insulin-induced by these compounds were first addressed. To begin with, the fluorescence emission of tyrosine amino acid was considered to assess the overall structure of insulin (Fig. 1A), and then the bis-ANS dye was used to evaluate the hydrophobic surfaces of insulin (Fig. 1B). Human insulin has four tyrosine amino acids in its structure, two residues located in the A-chain and two Tyrs placed in the B-chain of insulin. In the natively-folded structure of insulin, the amino acids tyrosine have unique positions that will change due to the small structural alterations in the integrity of insulin structure, causing possible changes in the fluorescence behavior of the fluorophore amino acid. In the current study, three different concentrations of the compounds (10, 20 and 30 μM) regarding the fixed insulin concentration (351 μM) were used. In general, in the presence of all three substances C1, C2 and C3, the fluorescence emission of tyrosine was increased with the increasing concentration of the compounds. However, the increase was the highest in the presence of the C2 while in the presence of the C3 analog, the lowest incremental changes were observed at all investigated concentrations (10, 20 and 30 μM). In comparison with C2 and C3 analogs, C1 compound, which is coumarin, had moderate increasing fluorescence emission effects on the human insulin molecule (Fig. 1A).

Given Fig. 1B, in the presence of the studied compounds, the emission of bis-ANS fluorophore has increased gradually by increasing concentrations of the analogs. When bis-ANS fluorescent dye interacts with hydrophobic surfaces of proteins, its fluorescence intensities would be increased. As shown (Fig. 1B), in the presence of all three investigated compounds and with different concentrations of them, the hydrophobic surfaces of insulin molecule increased. However, the increase in hydrophobic surfaces of insulin in the presence of methyl coumarin (C2) was dramatically more compared to the other counterparts (C1 and C3). Also, in the presence of 3-trifluoromethyl coumarin (C3), in agreement with Fig. 1A, the exposed hydrophobic surfaces of insulin were the lowest. Due to the hydrophobicity of analog C2 (Fig. S1C†), it can be inferred that the presence of a compound with a more hydrophobic moiety, strongly would affect the hydrophobic interactions of insulin and thus cause significant changes in insulin structure. Based on the previous attempts, insulin is known as an α-helix rich protein (44.5% α-helix, 15.7% β-sheet, 16.6% β-turn and 23.2% random coil).^31,37,38^ Here, using circular dichroism spectroscopy, secondary structure changes of insulin in the presence of the applied compounds at two pHs (pHs 2.0 and 7.4) were studied (Fig. 1C). In general, at acidic pH (2.2) secondary structural changes of insulin in the presence of any studied concentrations of C3 were not very significant (with a 3.8% transformation of α-helix to random coil at a 30 μM concentration of the compound). For C1 compound, with increasing the concentrations, major α-helix to random coil transitions in the structural integrity of insulin were seen. To be specific, at 30 μM of the C1, 44.5% and 15.7% of α-helical and β-sheet contents were reduced to 35.4% and 11.0%, respectively, while β-turn and random coil were increased to and 30.3% 23.6%. Similarly, by increasing the C2 concentration (to 20 μM), random coil and β-turn contents of the protein increased up to 37.8% and 27.5%, respectively. Interestingly though, by an increase in C2 concentration to 30 μM, the structure of the protein mainly transformed to irregular contents with a minor α-helical structures (17%) (Fig. 1C).

Principally, minimum CD spectra at 209 nm and 224 nm indicate α-helix contents while ellipticity at around 218 nm is a characteristic of β-sheet structure and minimum signals at ∼200 nm represent random coil structure.^39^ Because of the somewhat similarity between overall CD spectra at neutral pH (7.4), the signals were deconvoluted and represented as pie charts (Fig. 1D). It should be noted that the overall structural contents of insulin would be slightly changed by altering the pH of the medium.^37^ However, three main secondary structure contents of insulin did not significantly change by adding 30 μM of compounds into the solution (Fig. 1D). Under these conditions, insulin molecules are present in the hexameric form. Therefore, the data suggest that the compounds cannot induce secondary structural changes into the hexameric form of insulin at pH 7.4.

**Fig. 2:**
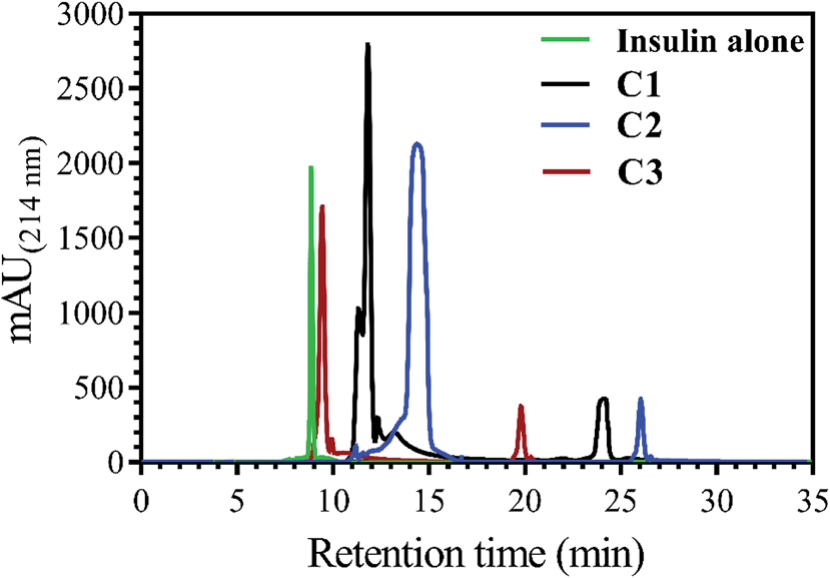
Comparing the RP-HPLC elution profile of the incubated insulin with the three applied compounds. Insulin (351 μM, under the explained acidic condition) was incubated with C1, C2 and C3 compounds (30 μM), separately, at 60 °C. The run time was set at 35 minutes while the running solution (H_2_O/ACN) with a linear gradient of increasing acetonitrile (24–60%) was chosen to elute the loaded insulin and corresponded compounds. Before conducting the experiment, insulin and all three compounds were separately loaded into the column for detecting their specific elution time (retention time). After ensuring that the chromatograms of all materials did not overlap with each other, the present setup was considered for RP-HPLC analyses of co-incubated insulin and the compounds.

In addition to measuring the hydrophobic surface area by bis-ANS dye, this property of insulin was also investigated using a reverse-phase high-performance chromatography column. In this assay, by increasing the hydrophobic levels in the surface of insulin, the elution time would be increased, as well (Fig. 2).

Insulin, without incubation as a control, has nearly 9.0 min retention time, while, when it was incubated with C1, C2 and C3 compounds, the observed retention time was seen to be 11.7, 14.9 and 9.7 min, respectively. In agreement with bis-ANS fluorescence study, the compounds had somewhat the same effects on exposing the hydrophobic surfaces of insulin.

### 3.2. Oligomerization behavior of insulin molecules in the presence of the C1, C2 and C3 compounds

In the presence of Zn ion, different pH strengths and insulin concentrations as well as in the presence of foreign factors such as nanoparticles and different polymers, insulin oligomerization would be changes through varied oligomeric states; hexamer, tetramer, dimer and monomer.^10,40–42^ In general, it is well accepted that lower oligomeric states of insulin are more susceptible to form fibrils than higher oligomeric states.^32^ In the current study, two pathways including dissociation of hexamer to monomer and oligomerization of monomer to higher-order oligomer were investigated in the presence of the compounds. Also, the fibril formation of both hexameric and monomeric forms of insulin was conducted, as well.

#### 3.2.1. Dynamic light scattering and size exclusion chromatography patterns of monomeric and hexameric forms of insulin in the presence of investigated compounds

**Fig. 3:**
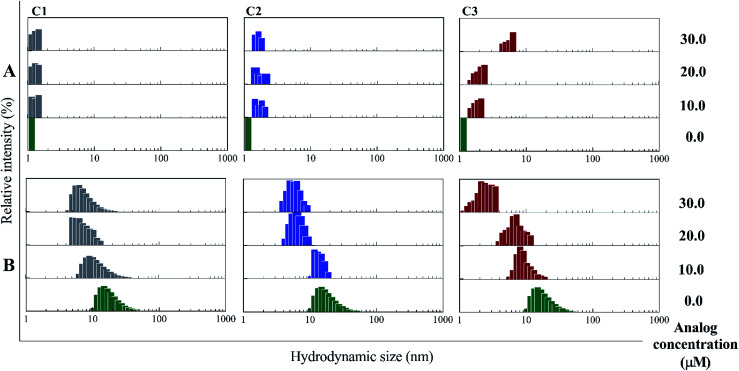
DLS studies of incubated insulin molecules with the compounds at pHs 2.2 and 7.4. Using different concentrations of C1, C2 and C3 (10, 20 and 30 μM) the oligomerization patterns of insulin were studied. (A) shows this experiment at acidic condition (pH 2.2), in which insulin is mainly present as monomer at the beginning of this condition. (B) stands for the study under neutral condition (pH 7.4) wherein the hexameric state of insulin is the starting form of the protein. Acidic and neutral conditions were studied at 60 °C and 37 °C temperatures.

Aforesaid, oligomerization assays were performed in both acidic (Fig. 3A) and neutral (Fig. 3B) solutions to provide suitable conditions for the formation of monomeric and hexameric forms at the initial stage of the experiment. According to Fig. 3A, C1 and C2 compounds had little effect on changing the oligomerization patterns of the insulin monomer, while C3 analog, by increasing the concentration, induced monomeric insulin molecules to higher oligomeric states. According to Fig. 3B, these C3-induced oligomers are smaller than the hexameric form. Not like the acidic state, when DLS patterns of insulin were investigated at the neutral condition (Fig. 3B), the presence of all three compounds in different concentrations progressively induced the dissociation of hexameric forms of insulin to lower oligomeric states. This change that mediated by C3 analog was greater than the changes in the presence of the other counterparts (C1 and C2), indicating the strong effect of trifluoromethyl group on the dissociation of the hexameric form of insulin.

**Fig. 4:**
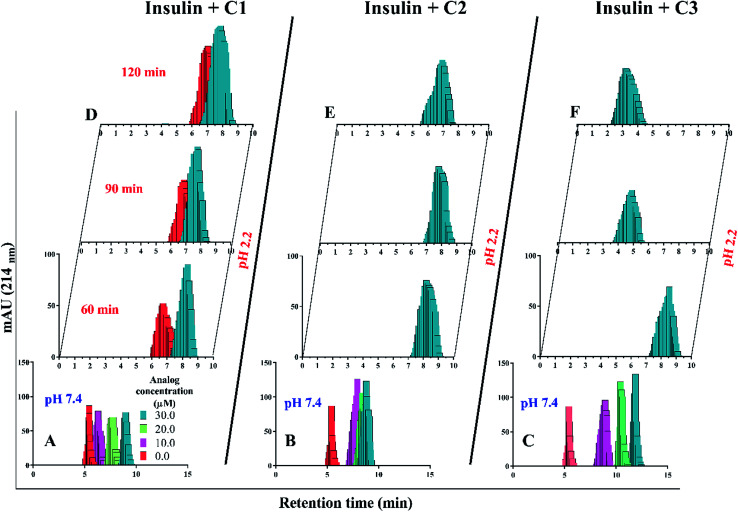
SEC studies of insulin molecules in both monomeric (pH 2.2) and hexameric (pH 7.4) states in the presence of the compounds. The compounds-mediated changes of hexameric insulin oligomerization (A–C) were studied under neutral condition (pH 7.4), while the changes in monomeric states of insulin using the compounds were analyzed at acidic condition (D–F). The study under acidic and neutral conditions was carried out at 60 °C and 37 °C temperatures.

Retrospectively, the patterns of insulin oligomerization were investigated using a gel filtration column (size exclusion chromatography, SEC) in the two defined solutions. Fig. 4A–C correspond to the changes of insulin hexamer in neutral condition and Fig. 4D–F have to do with the alteration of the monomeric population of insulin in acidic conditions. In the neutral pH, in the presence of all three compounds C1, C2 and C3 hexameric states of insulin gradually shifted to monomeric forms with increasing concentration of the compounds. This effect was observed to be more notable when C3 analog was incubated with insulin (Fig. 4A–C). Looking through the effect of the compounds on monomeric insulin, to intensify the oligomerization changes in the acidic state and to the better detection of the output signal by the chromatography, insulin was incubated in this condition with nothing but 30 μM concentration of the compounds and gel filtration was examined at different time frames. In general, when the concentration of the compounds was increased, the insulin molecules shifted from the hexamer states to the ones with a smaller oligomerization ratio. These changes, compared with C1 and C2 compounds, consistent with DLS data, are greater for C3 analog (Fig. 4C). As the concentrations of coumarin and its analogs were increased, the observed changes in the hexameric states were amplified. To investigate the effect of the presence of the compounds on the rate of insulin aggregation/fibrillation, the changes of insulin monomeric form at a constant concentration of insulin (351 μM) and the compounds (30 μM) were studied. Also, the incubation temperature was considered to be 60 °C and the mixtures were injected into the gel filtration column after 60, 90 and 120 minutes (the time intervals were selected according to kinetic study that will be discussed later). The changes in insulin monomeric states to higher oligomer in the presence of coumarin (C1, Fig. 4D) at 60 to 120 min time incubations somewhat inhibited in comparison with those changes related to insulin alone, showing that coumarin can modulate insulin aggregation during the time frame. Similarly, C2 was slightly successful to reduce the rate of insulin aggregation, while in the case of C3 analog, in the studied concentrations, the protein communities generated from insulin monomers were observed to be dramatically larger than the control experiment, indicating that the analog was able to increase insulin aggregation (Fig. 4E and F).

### 3.3. Studying the effect of the synthetic compounds on insulin stability

**Fig. 5:**
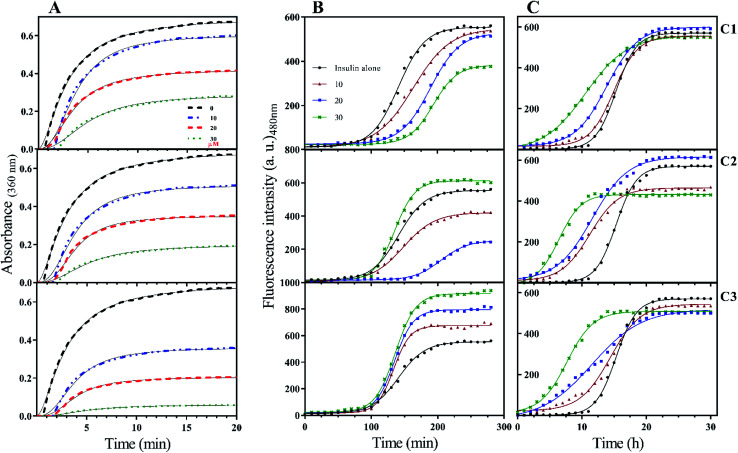
Kinetic of insulin stability under reducing, acidic and neutral conditions. (A) indicates the kinetic of DTT-mediated insulin aggregation in the presence of different concentrations (10, 20 and 30 μM) of the compounds. This experiment was carried out at 43 °C. Using ThT dye as a fibril growth fluorophore detector dye, the fibrillation process of insulin at acidic (B) and neutral (C) environments were analyzed. (B and C) experiments were done at 60 °C and 37 °C temperatures, respectively. All spectra represent an average of at least three independent experiments.

To the ideal and proper function of a protein or peptide, in addition to their structure, their stability must also be in a favorable range. In the field of therapeutic recombinant proteins, many efforts have been made in search of increasing the stability of proteins under environmental-chemical and physical stresses. Therefore, it is very important to investigate the effect of the studied compounds on insulin stability. Insulin has three disulfide bonds, two bonds are located between A- and B-chain while one disulfide bond is positioned within A-chain as an intrachain disulfide bond. With the reduction of any of these bonds, the overall structure of insulin would be destroyed and consequently precipitated. In the current study, DTT-induced disulfide reduction was used for examining the overall effects of the studied compounds on the kinetic of insulin precipitation (Fig. 5A). This experiment was performed at a temperature of 43 °C. According to Fig. 5A, the kinetic rate of insulin aggregation in the presence of all three compounds is generally lower than the control. So that the data obtained from the study of different concentrations show that this inhibition of aggregation is dependent on the concentration of synthetic compounds (concentration-dependent manner). Among the three compounds C1, C2 and C3, the most inhibition was observed in the presence of the later analog. Similarly, analog C2 had a more delayed effect on insulin aggregation compared with C1.

When it comes to the stability of amyloidogenic proteins or peptides, it is important to measure the fibrillation rate of the sample using ThT fluorophore. After binding to protein fibrils, the highest absorption and emission wavelengths of the dye shift to 450 nm and 484 nm, respectively. In this study, the rate of insulin fibril formation in the presence of three compounds in both acidic (Fig. 5B) and neutral (Fig. 5C) solutions was investigated. At acidic condition, with increasing the concentration of coumarin (C1), the rate of insulin fibril formation gradually decreased, while in the presence of C2 analog, the inhibition of the process was observed only at concentrations of 10 and 20 μM while at 30 μM, the rate of fibril formation increased. Interestingly though, when insulin fibrillation was examined in the presence of different concentrations of C3 analog, the rate of fibril formation slightly increased by increasing the concentrations (10, 20 and 30 μM). Generally speaking, the rate of insulin fibrillation in the neutral environment is slower than in acidic conditions.^32^ Studies on the formation of insulin fibril in the neutral environment and the presence of the studied compounds showed that the presence of all three compounds increased the kinetics of insulin fibrillation, in which the effect of C3 analog was more pronounced in comparison with C1 and C2 compounds. All of those improvements were concentration-dependent manner (Fig. 5C). For more details, lag times of different rates of the fibrillation were calculated and presented in Table 1.

**Table 1:** Different lag times of insulin fibrillation in the presence of the various amount of the compounds

	Concentration of the compounds (μM)	Lag time	
		pH 2.0 (min)	pH 7.4 (h)
Insulin	0	96	12.3
	10	105	11.2
Insulin + C1	20	137	8.3
	30	154	3.8
	10	99	6.2
Insulin + C2	20	157	5.6
	30	102	3.4
	10	101	7.5
Insulin + C3	20	94	4.2
	30	90	3.1

### 3.4. Morphological studies of preformed fibrils

**Fig. 6:**
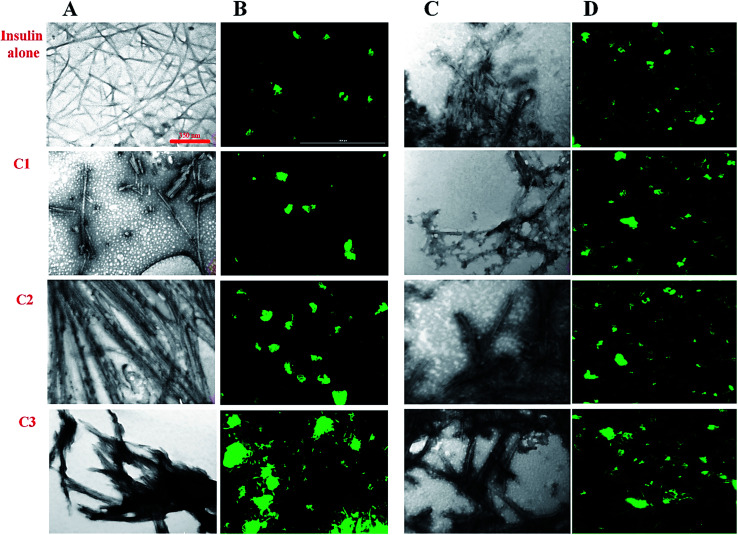
Morphological assessments of preformed insulin fibrils in different conditions. (A and B) respectively, indicate the morphology of insulin fibrils at acidic condition (pH 2.2) using TEM and fluorescence microscopies whereas (C and D) show the fibril architects at pH 7.4. The fibrils were performed in the maximum concentration of the compounds (30 μM). TEM microscopy show length and more resolution of contact between fibrils while fluorescence microscopy indicates the overall bundle of fibrils. For TEM images (A and C), the scale bars are 350 nm and for fluorescence micrographs (B and D) they are 1000 μM.

In previous studies, the relationship between the final architecture of fibrils and their ability for the destruction of cell membranes has been studied and some mechanisms were proposed.^35,36^ In this study, transition electron microscopy (TEM), fluorescence microscopy, Raman and FTIR assessments were used to investigate the effect of the studied compounds on the insulin fibril morphology. Then, the cytotoxicity of the fibrils was studied. For this purpose, the compounds and insulin were sampled at a 30 μM concentration of the compounds and 351 μM concentration of insulin for 6 and 30 hours to ensure fiber formation at acidic and neutral conditions, respectively. We used a high concentration of the compounds (30 μM) as representative of all studied concentrations. According to the TEM and fluorescence images (Fig. 6), in the presence of coumarin (C1), the short fibrils of insulin with negligible lateral interactions were observed. However, in the presence of 7-methyl coumarin (C2) which has a hydrophobic moiety, the fibrils are formed as long strands with high lateral connections. Interestingly, the insulin fibrils formed in the presence of 3-trifluoromethyl coumarin (C3) were observed as short fibrils with abundant lateral linking (Fig. 6A and B).

Looking generally, fluorescence microscopic images show bundle (lateral interaction between fibrils) and the population of fibrils in each sample. For fluorescence microscopy, the preformed fibril samples were first incubated in 20 μM ThT fluorophore and then spread uniformly on glass slides for 30 minutes in a dark room. The slides containing the sample were then imaged by fluorescence microscope. According to the results (Fig. 6B), in the presence of C3 analog, the fibrils bundles are much close to each other. However, the preformed fibrils containing C2 analog also have significant communities, nevertheless, those fibrils aggregates in the presence of coumarin (C1) were represented as small light clots indicating the less accumulation of fibers. In general, in harmony with the TEM data, these images showed that C3 and then C2 have the greatest effect on the formation of a bundle of fibrils. Interestingly, both fibrils and bundles of fibrils generated in the presence of the compounds at neutral conditions (Fig. 6C and D) showed somewhat significant differences, indicating more thick bundles in the presence of C2 and C3 analogs. However, these fibrils were not very significant in term of longitudinal growth.

**Fig. 7:**
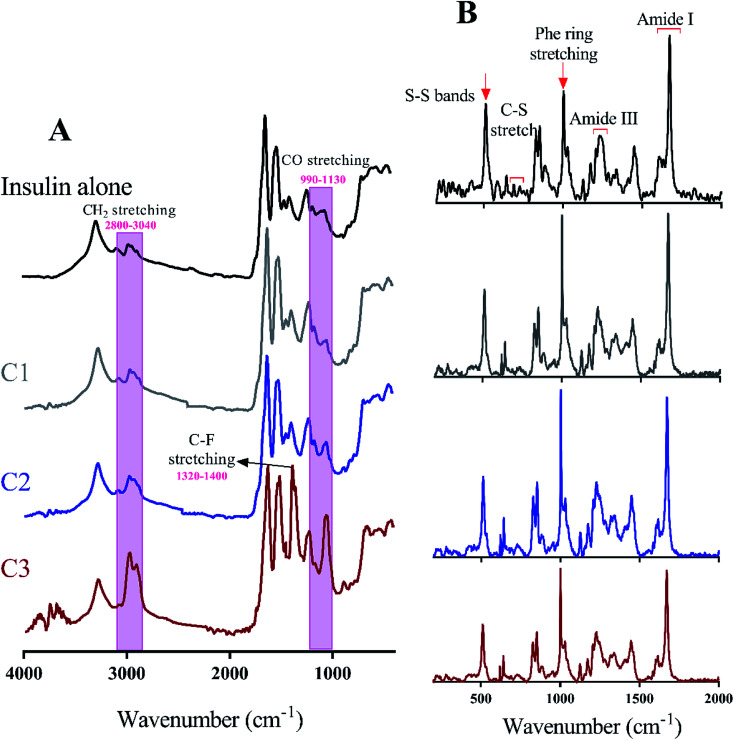
FTIR and Raman spectroscopies of the compounds-mediated preformed insulin fibrils. (A) shows FTIR spectra (4000 to 400 cm^−1^) of preformed insulin fibrils in the presence and absence of the compounds. Likewise, (B) indicates Raman spectra (200–2000 cm^−1^) of the generated insulin fibrils at similar conditions to the (A).

Many recent studies have been shown that proteinous fibrils with a specific secondary structure can have a greater effect on cell membrane destruction and cytotoxicity.^35,43^ Of the most common methods to assess the secondary structure of proteinous fibrils the use of Raman and FTIR spectroscopies are specifically appreciated. In this study, both methods were used for better judgment of the secondary structures of preformed insulin fibrils in the presence of the studied compounds. In the case of Raman and FTIR spectroscopies, these methods are sensitive to the functional groups of the fibril structures, and any structural changes that cause the displacement of these groups can be observed by those spectroscopies. Raman spectroscopy, also, can show the formation and breakage of disulfide bonds within the fibrils. Due to the more or less longitudinal growth similarity of the preformed fibrils under neutral solution (Fig. 6C and D), the further structural analyses were conducted just for those generated fibrils in the acidic environment. Given Fig. 7A, the fibrils studied by FTIR spectra are significantly different from each other in the two regions of 990–1130 cm^−1^ and 2800–3040 cm^−1^ which respectively indicate the difference in stretching of CO and CH_2_ in the structure of the fibrils. The changes in CO stretching can be due to deviations in the protein backbone torsion in the mediated C2 and C3 fiber structures. On the other hand, the fibril formed in the presence of C3 analog has a signal at 1320–1400 cm^−1^ indicating C–F stretching which is possible evidence of the existence of this analog in the structure of the fibers. According to Raman spectroscopic studies (Fig. 7B), no significant differences were seen in terms of the position of the disulfide bonds, Phe ring stretch signals and the amide III region, supporting the protecting role of the compounds on disulfide bridges.

**Fig. 8:**
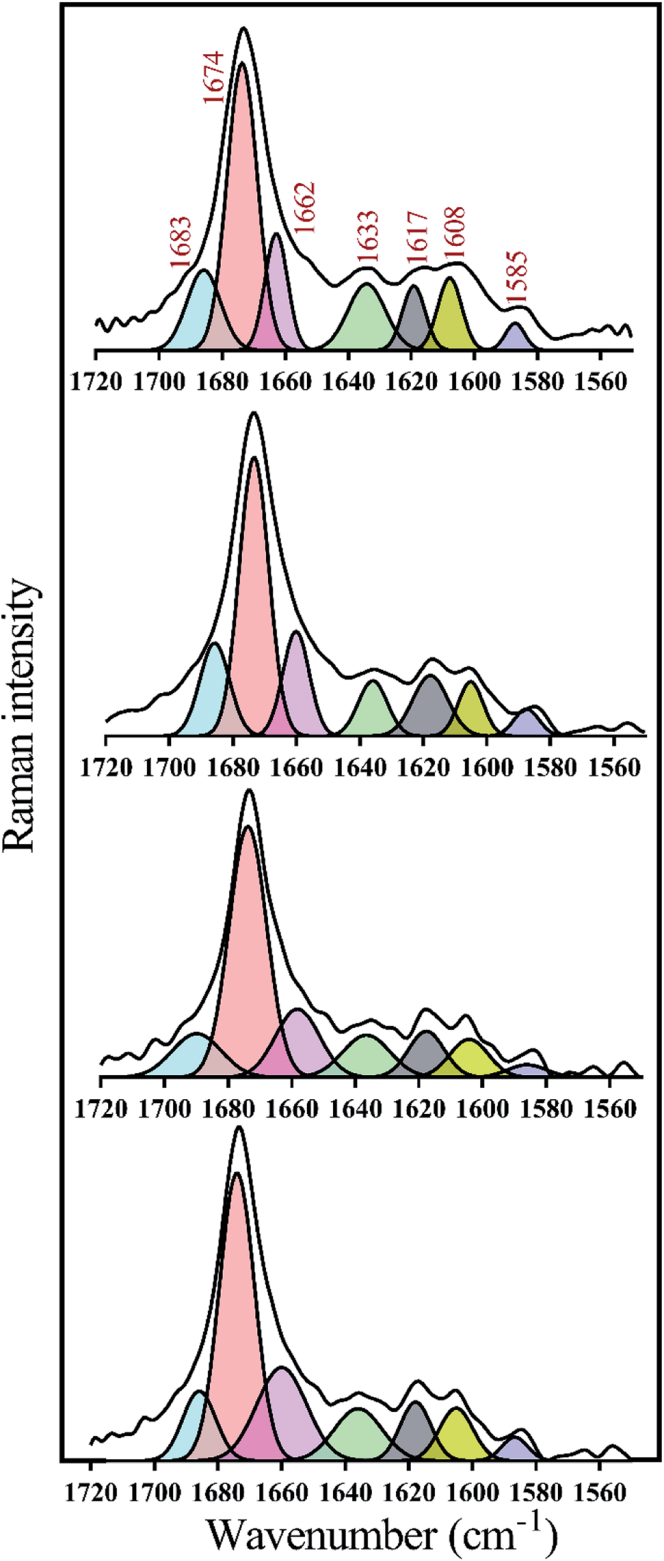
Deconvolution analyses of Raman spectra in the amide I region. The deconvolution analyses of amide I (1720 to 1560 cm^−1^) region which represents secondary structure contents of preformed insulin fibrils in different conditions were indicated. These preformed fibrils were generated at 30 μM concentration of the compounds. According to the previous study,^44^ Raman signals at 1683, 1674, 1662 and 1633 wavenumbers correspond to disordered, β-sheet, α-helix and disordered/vibronic coupling bands, respectively. Also, 1617, 1608 and 1585 show ring modes of Phe and Tyr residues.

To take a deeper look at the structure of the fibrils, the extended amide I regions which are corresponding to secondary structure contents of the fibrils were analyzed, separately (Fig. 8 and Table 2). In general, with the formation of insulin fibrils, the percentage of β-sheet content in the structure of proteins would be increased,^31,32,41^ which can be seen in all the generated fibrils (Table 2). For more details, by transforming insulin molecules to ordered fibrils, 44.5% of the α-helical contents reduced to 15% whereas 15.7% of the β-sheet structure increased to 57%. Moreover, overall disordered contents of generated fibrils have also increased to 16%. When insulin fibrillation was done in the presence of C1 compounds, in comparison with C1 treatment, more helical structures were converted to β-sheet although the overall disordered structures were slightly reduced. β-Sheet contents of formed fibrils were at the maximum level when C2 compound was used for the generation of the insulin fibril. In comparison with C1 and C2 compounds, those insulin fibrils which formed in the presence of C3 compounds showed intermediate structural characteristics. It is worth mentioning that in all of the fibers, the structures around the phenyl rings had almost the same changes. Overall, during insulin fibrillation, C2 was more β-sheet inducer than C1 and C3.

**Table 2:** Deconvolution of amide I region to assess secondary structural alteration of the fibrils^a^^44,45^

	Disordered	β-Sheet	α-Helix	Disordered/vibronic coupling bands	Ring modes of Phe and Tyr		
Wavenumber (cm^−1^)	1683	1674	1662	1633	1617	1608	1585

**Area%**							
Insulin	9	57	15	7	4	5	3
Insulin + C1	8	62	12	5	7	4	3
Insulin + C2	6	68	10	6	5	4	1
Insulin + C3	7	63	14	7	4	3	3

a The deconvolution was done by OriginLab 2018 software.

### 3.5. *In vitro* cytotoxicity of co-incubating insulin fibrils and the applied compounds

**Fig. 9:**
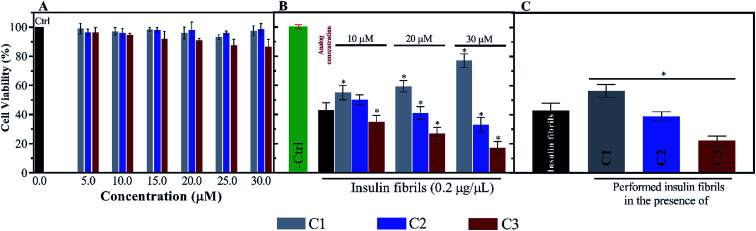
Neural cell (N2A) viabilities in the presence of C1, C2 and C3 as well as exposing the cells with preformed insulin fibrils and the compounds. (A) indicates cytotoxic effect of the compounds at five concentrations (5.0, 10.0, 15.0, 20.0, 25.0 and 30.0 μM). (B) displays the N2A cytotoxic effects of the coincubation of preformed insulin fibrils and the compounds at three indicated concentrations. (C) represents the toxicity of the only preformed insulin fibrils in the presence of the compounds. All experiments were replicated 5 times to minimize well-to-well variations.

For further investigation of the insulin fibrils, their cytotoxicity was evaluated using N2A neural cells. Depending on the interaction between the fibrils and the surface of the cell membrane, the toxicity of the fibrils can be different. According to Fig. 9A, the toxicities of the compounds were initially investigated. Given the figure, the compounds (mainly C1 and C2) at the six indicated concentrations did not have significant toxicities for the cells. Nonetheless, among the three compounds C1, C2 and C3, the later analog showed more toxicity at those concentrations higher than 20 μM. However, the coincubation of preformed fibrils and the compounds with the cells showed comparable toxicities (Fig. 9B). Exposing the cells with C1 and insulin fibrils indicated that by increasing the compound concentration in the medium, cell viability increased up to 70%. On the contrary, the coincubation of insulin fibrils and the analogs of coumarin showed increased cell death with the increase in the analogs concentration. This effect of C3 was in the highest amount. The cytotoxic effects of preformed insulin fibrils showed interesting results (Fig. 9C). The data indicated that those fibrils preformed in the presence of C1 had the least toxic effect on the cells while fibrils generated in the presence of C2 and C3 had more toxic effects in comparison with the control sample. The plot showed that C3-mediated insulin fibrils had the most toxic effect on the N2A cells.

## 4. Discussion

So far, many studies have been conducted to ask how the stabilization of pharmaceutical proteins or peptides can be achieved. In this paper, using coumarin as a bio-safe plant-derived phenolic compound and two types of its analogs that are hydrophobic and hydrophilic, we tried to study insulin fibrillation and cytotoxicity. Although in our previous study the consequence of positive charges on the process of insulin fibrillation was observed to be inhibitory effect, in this study it was showed that the introduced positive charges on the phenolic ring have an opposing effect on the process.^5^ Considering that each of the studied compounds C1 and C2 had a significant structural effect on insulin (Fig. 1) and, on the other hand, C3 had a minor effect on the unfolding of insulin, it can be concluded that in an acidic environment which is suitable for monomeric form of insulin (Fig. 3 and 4), the presence of phenolic groups (C1) and the hydrophobic nature of 7-methyl coumarin (C2) can interact with the insulin molecule and affect the natively-folded structure of this hormone. From a mechanistic point of view, looking through all previously studied fibril-inhibitor compounds on insulin, quercetin is the most similar compound to coumarin.^16^ For explaining the inhibitory effects of compounds such as these, the factors presented so far include aromatic stacking between phenolic rings and hydrophobic residues, hydrophobic interactions, and hydrogen bonding. It also has been believed that some of the examined compounds can strongly bind to partially unfolded insulin and stop structural changes that lead to amyloid fibrillation.^42,46,47^ It has also been studied that there are several partially unfolded insulin intermediates, among which there is only a certain intermediate that is very vulnerable to fibrillate.^38,48^ As a result of which intermediate the substance used in a study can convert insulin to, it may have a strong or weak inhibitory effect. It is worthy to emphasize that C1 and C2-mediated structural changes on insulin were occurred only in the acidic condition (pH 2.0), nonetheless, in the neutral environment (pH 7.4), no significant changes were observed, which indicate that the studied compounds cannot alter the structural integrity of the hexameric state of insulin. Following this result, in another study, different states of insulin oligomerizations have also been considered as an important factor in explaining the mechanism of the inhibition of process.^49^ Oligomerization studies (Fig. 3 and 4) showed that the selected compounds can separate the hexamer state of insulin into smaller oligomers. Also, in the acidic solution, analog C3, can attack insulin molecules and make high molecular oligomers (*cf.*Fig. 3A and 4C). Because the effect of C3 in an acidic environment on insulin oligomerization was greater than analog C2, it can be proposed that electrostatic interactions (owing to –CF_3_ group) between C3 and insulin molecules are stronger than hydrophobic interactions between the two molecules (Fig. 3A). Similar to this result, Shinde *et al.*, previously studied that using negatively charged *p*-sulfonatocalix[4/6]-arene macrocyclic hosts (SCX4/6), insulin fibrillation can be attenuated.^50^ This effect can also be interpreted in Fig. 3B. This figure shows that the role of electrostatic interactions and the presence of negatively charged ions have a greater effect on the degradation of insulin hexamer to smaller states. On account of Table S1,† this result could be due to the electrostatic attack of the fluoride atoms (–CF_3_) in analog C3 on the positive Zn ions positioned on the central of insulin hexamer. After this attack, the fluorine atoms can be replaced with the imidazole in the side chain of His^B10^. Besides, our data from Fig. 3 and 4 suggest that phenolic rings and hydrophobic groups also have a minor role in the destruction of the hexameric oligomer of insulin molecules indicating that organizing hexamer state is strongly depends on coordination between His^B10^ and two central Zn ions. Furthermore, kinetic studies (Fig. 5) suggest that during insulin fibrillation at acidic conditions, in addition to the oligomerization of insulin, the structural changes of the molecule also affect the rate of insulin fibril formation (Fig. 5A). However, in the neutral pH, considering that the compounds did not cause structural changes in the insulin molecule, it can be concluded that the kinetic of the fibrillation (Fig. 5C) is mostly due to the different state of insulin oligomerization (*cf.*Fig. 3, 4 and 5C). Moreover, the studies of insulin aggregation in the presence of reducing agent DTT showed that the compounds may be able to provide support for disulfide bonds, thereby, preventing rapid DTT-mediated insulin aggregation (Fig. 5A). Our data from Fig. 6 suggest that the presence of hydrophobic interactions between the additives and insulin (C2) are more involved in the longitudinal growth of fibrils, while the presence of electrostatic interactions causes the lateral interaction of the fibrils and the formation of short length bundles. A closer look at the previous studies will confirm the current conclusion. The proposed inhibitory mechanism of benzene and toluene as hydrophobic compounds on insulin fibrillation has been shown that the generated fibrils were short and amorphous.^42^ In general, bearing in mind that the coumarin compound has two hydrocarbon rings, it can be suggested that the presence of these rings without any chemical group is effective in the formation of short fibrils and affects the kinetics of fibril formation. The given results from Fig. 7, 8 and Table 2 propose that the secondary structures of the preformed fibrils in the presence of the additives have not significant differences, indicating that the observed differences in the fibrils cytotoxicity are due to the overall communities and the length of the proteinous fibrils. So that fibrils with a short length and abundant lateral-links have the greatest toxicity effect on the nerve cells (*cf.*Fig. 6 and 9C).

**Scheme 2:**
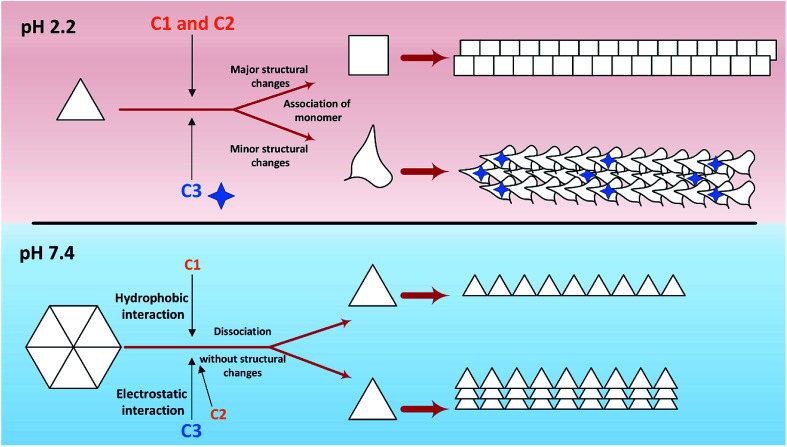
Graphical abstract extracted from the obtained results. The red background shows the effects of three investigated compounds at acidic pH on insulin fibrillation, while the blue background indicates these effects at pH 7.4. At pH 2.2, the association of insulin molecules to higher oligomeric states along with the unfolding of the molecules taken place. Two series of unfolded molecules produce two morphologically different fibrils. The result from Raman analysis shows that C3 analog can participate structurally in the fibrils. Nevertheless, at neutral pH, only the dissociation of insulin hexamers without any CD analyses detectable structural changes occurs. All preformed insulin fibrils produced at this pH and in the presence of the investigated compounds were observed in somewhat similar in longitudinal patterns while lateral interactions were observed to be more in the presence of C2 and C3 analogs.

Putting all the results together, a general schematic (Scheme 2) can be suggested.

Aforementioned, in acidic environments, compounds C2 and C3 are capable to alter the native structure of insulin and create the intermediates of insulin that are vulnerable to fibrillate (Scheme 2 and Fig. 5). Our previous study^48^ showed that these intermediate can govern the overall pathway of the fibrillation. Eventually, these effects can cause long fibers in the presence of C2 and short fibers by using C3 analog. The evidence from Raman spectroscopies showed that analog C3 structurally contribute to the fibrils architecture. Using chloride ions as negative additives, a similar result has been proven in a previous study.^51^ Also, in neutral solution, the most important role of C2 and C3 analogs was to separate insulin hexamers into monomers (*cf.*Fig. 2–4 as well as Scheme 2). After the dissociation of hexameric insulin to the monomeric states, they undergo to make fibrils in the stressogenic environment, more rapidly. By and large, the results of this study show that the presence of phenolic rings and their effects on both kinetic and morphology of the fibrils strongly depend on the pH of the investigated solution. In the presence of the chemical candidates, altering the oligomerization states of insulin in physiological pH dramatically changes the kinetics of insulin fibrillation but has no significant effects on the morphology of the generated fibrils. Also, it was showed that cytotoxicity of the fibrils also depends on the length and degree of lateral-links between the fibrils. However, further study is needed to find out this question; why those insulin intermediates under acidic condition (detected by CD signals) produced rather similar mature fibrils (detected by Raman and FTIR studies)?

In brief, using several physicochemical and *in vitro* cytotoxicity methods we found that for studying chemical compounds on the stability of proteins, pH of the considered environment is critically important. In this study, our selected compounds had different effects on the fibrillation of insulin, both kinetically and morphologically. Also, not only does the secondary structure of fibrils play an important role in cytotoxicity (rely on the previous studies),^52–54^ here, we found that overall organization, lateral connection and length of the fibrils are significantly important in the effect of the aggregates on cell membrane disruption.

## 5. Conclusion

Information on the effect of additives can be a promising solution for bio-engineer researchers in the field of therapeutic proteins and peptides to improve the stability of such important molecules. Based on the obtained results, for studying the effect of additives on the fibrillation of insulin or even other amyloidogenic proteins, it is important to consider the different modes of oligomerization. So that some additives such as 3-trifluoromethyl coumarin (C3) without significantly altering the structure of insulin can change the kinetic of fibrillation by changing its oligomerization states. As a result, changing the rate of insulin fibrillation by altering its structure will not always the only way. Furthermore, it is important to note that the cytotoxicity of insulin fibrils can be due to the general arrangement of the fibrils even the fibrils with more or less similar secondary structures.

## Conflicts of interest

The authors declare no conflict of interest.

## Supplementary Material

RA-010-D0RA07710K-s001
