# Supplementary material for: Inhibitory effect of coumarin and its analogs on insulin fibrillation /cytotoxicity is depend on oligomerization states of the protein
Source: RSC Adv. 2020 Oct 16;10(63):38260–74. doi: 10.1039/d0ra07710k (PMC9057281; doi:10.1039/d0ra07710k)
Supplement: RA-010-D0RA07710K-s001 [file RA-010-D0RA07710K-s001.pdf]

Electronic Supplementary Material (ESI) for RSC Advances.
This journal is © The Royal Society of Chemistry 2020

Inhibitory Effect of Coumarin and Its Analogs on Insulin Fibrillation /Cytotoxicity Is Depend on Oligomerization States of the Protein

Mohsen Akbarian[a,b], Ehsan Rezaie[a*], Fatemeh Farjadian[b], Zahra Bazyar[c], Mona Hosseini-Sarvari[c], Ehsan Malek Ara[d], Seyed Ali Mirhosseini[d], Jafar Amani[d]

[a] Molecular Biology Research Center, Systems Biology and Poisonings Institute, Baqiyatallah University of Medical Science, Tehran, Iran.

[b] Pharmaceutical Sciences Research Center, Shiraz University of Medical Sciences, Shiraz, Iran.

[c] Department of Chemistry, Shiraz University, Shiraz, Iran.

[d] Applied Microbiology Research Center, Baqiyatallah University of Medical Sciences, Tehran, Iran

[*]Correspondence: email address

Ehsan Rezaie, Molecular Biology Research Center, Systems Biology and Poisonings Institute, Baqiyatallah University of Medical Science, Tehran, Iran. P.O. Box 19395-5487. Tel: +98 21 82454555, Fax: +98 21 83062555. E-mail: rezaie.ehs@gmail.com.

Supporting Methods

Preparation of coumarin analogs

The analogs of coumarin were synthesized as our previously published method. In brief, in a Pyrex tube, a mixture of 2*H*-Chromen-2-one (1.0 mmol), Au@ZnO nanoparticles (4.0 mg), K_2CO_3 (1.5 mmol) and $CHCl_3$ (3.0 mL) was carried out under argon atmosphere and 11 W blue LEDs at room temperature for 24 h. When the reaction was complete, it was centrifuged to remove the catalyst and the mixture was purified by column chromatography on silica gel (Petroleumether: Ethyl acetate) to give the desired product. The product was characterized with ^{1}H NMR, ^{13}C NMR, IR and CHNS[2]. [1, 2]

Supporting Results

Characterization of the investigated compounds in this study

Among the studies of biological evaluations of chemical compounds, it is common to first assess the stability of the compounds during the study. Therefore, herein, the stability of coumarin and its two analogs of 7-methyl coumarin and 3-trifluoromethyl coumarin was first considered. According to **Figure S1A**, the absorption of the compounds was initially recorded at wavelengths between 250-450 nm. The λmax absorptions of compounds C1, C2 and C3 were observed to be 258 nm, 298 nm and 327 nm, respectively. Using these maximum absorption points, the emission spectra of the chemical candidates were also recorded. According to **Figure S1B**, the emission spectra were recorded in the range of 300 nm to 700 nm. Maximum emission wavelengths of 426 nm, 453 nm and 493 nm were observed for the C1, C2 and C3 compounds. In the next section, reverse-phase high-performance liquid chromatographic (RP-HPLC) measurements were used to study the hydrophobicity of the applied compounds. In RP-HPLC analysis, materials with more hydrophobicity are eluted later than hydrophilic compounds through the chromatographic column (C18). According to **Figure S1C**, C3 eluted faster than the other two compounds through the column, indicating the least hydrophobicity or the highest hydrophilicity among the other counterparts. Besides, the data showed that C2 analog has the highest amount of hydrophobicity, while C1 is moderate in this respect. Linking this result with **Scheme 1**, it can be concluded that the presence of methyl group in coumarin structure would increase hydrophobic property while the presence of trifluoromethyl group can reduce this characteristic. On account of data from **Figure S1B**, those maximum emission wavelengths were used to investigate the stability of

coumarin and its analogs at different time intervals. Based on the results from **Figure S1D**, C1 compound had the lowest stability, while the highest resistance to degrade was observed for C2. The stability of C3 is characterized between these two compounds. To determine the charge of the studied compounds, zeta potential measurements were done. As shown (**Table S1**) the pH of the considered experiments had notable effect on obtained zeta potential. Between all conditions, C3 analog at pH 7.4 showed negative zeta potential. C1 and C2 compounds in the both pHs of 2.2 and 7.4 showed positive zeta potential.

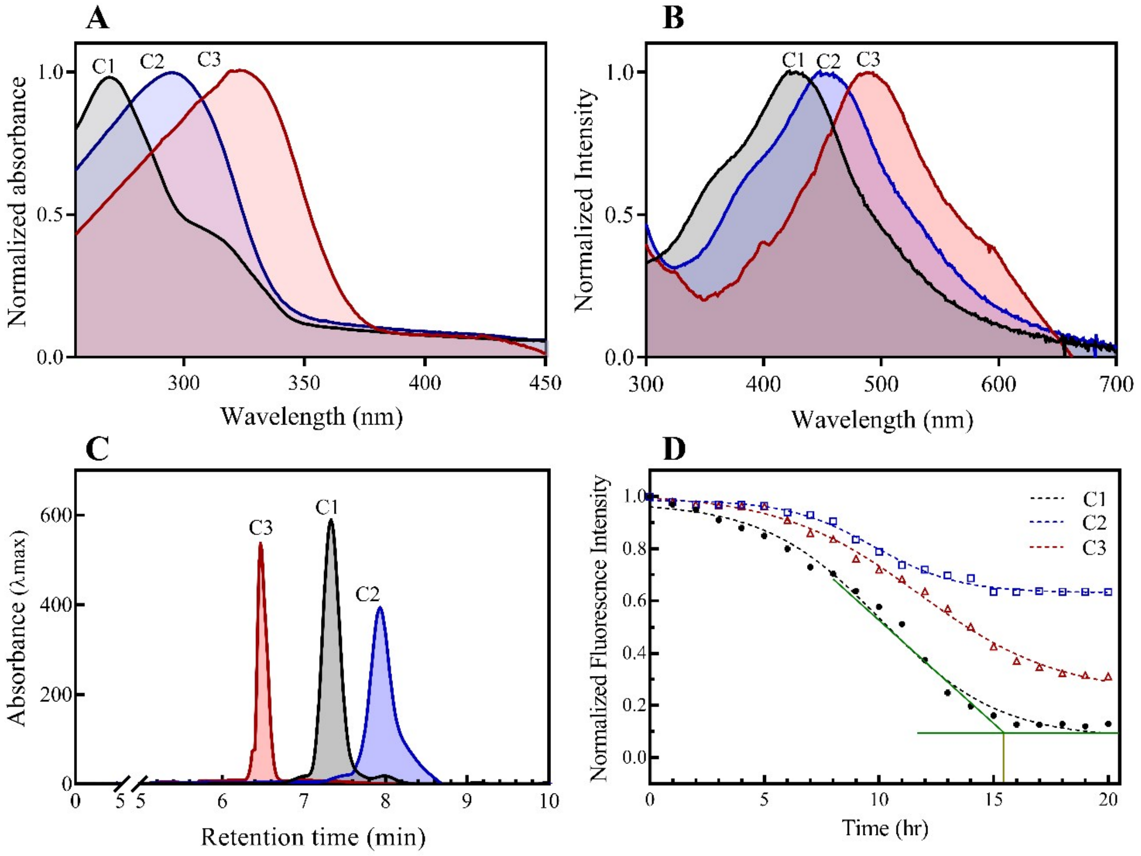

Figure S1. Some characterizations of the considered compounds in this study.

A and **B** respectively show absorbance and emission spectra of C1, C2 and C3 compounds, while **C** indicates RP-HPLC chromatograms of them. **D** displays quenching of the emission points (λmax) of the compounds in different time intervals (the stability of the compounds was studied in the acidic-fibrillogenic condition which is harsher than neutral pH condition).

Table S1. Zeta potential measurements of the applied compounds.

Compound	Zeta potential (mV)	
	**pH 2.2**	**pH 7.4**
**C1**	+3.3 ± 0.5	+1.1 ± 0.5
**C2**	+5.5 ± 1.2	+1.5 ± 0.8
**C3**	+11.2 ± 1.1	-7.4 ± 1.4

1. Z. Bazyar and M. Hosseini-Sarvari, *The Journal of organic chemistry*, 2019, 84, 13503-13515.
2. Z. Bazyar and M. Hosseini-Sarvari, *Organic Process Research & Development*, 2019, 23, 2345-2353.
